# Time-Resolved Transcriptomics and Bioinformatic Analyses Reveal Intrinsic Stress Responses during Batch Culture of *Bacillus subtilis*

**DOI:** 10.1371/journal.pone.0027160

**Published:** 2011-11-08

**Authors:** Evert-Jan Blom, Anja N. J. A. Ridder, Andrzej T. Lulko, Jos B. T. M. Roerdink, Oscar P. Kuipers

**Affiliations:** 1 Department of Genetics, University of Groningen, Groningen Biomolecular Sciences and Biotechnology Institute, Groningen, The Netherlands; 2 Johann Bernoulli Institute for Mathematics and Computing Science, University of Groningen, Groningen, The Netherlands; 3 Kluyver Centre for Genomics of Industrial Fermentation, Delft/Groningen, The Netherlands; Loyola University Medical Center, United States of America

## Abstract

We have determined the time-resolved transcriptome of the model gram-positive organism *B. subtilis* during growth in a batch fermentor on rich medium. DNA microarrays were used to monitor gene transcription using 10-minute intervals at 40 consecutive time points. From the growth curve and analysis of all gene expression levels, we identified 4 distinct growth phases and one clear transition point: a lag phase, an exponential growth phase, the transition point and the very clearly separated early and late stationary growth phases. The gene expression profiles suggest the occurrence of stress responses at specific times although no external stresses were applied. The first one is a small induction of the SigB regulon that occurs at the transition point. Remarkably, a very strong response is observed for the SigW regulon, which is highly upregulated at the onset of the late stationary phase. Bioinformatic analyses that were performed on our data set suggest several novel putative motifs for regulator binding. In addition, the expression profiles of several genes appeared to correlate with the oxygen concentration. This data set of the expression profiles of all *B. subtilis* genes during the entire growth curve on rich medium constitutes a rich repository that can be further mined by the scientific community.

## Introduction

*Bacillus subtilis* belongs to the Gram-positive Firmicutes and has been investigated for more than a century. Moreover, *Bacillus species* have also been used in biotechnology as industrial production strains for a long time. *B. subtilis* serves as a model organism and is considered a reference for cell differentiation and adaptation. This model status makes it one of the most extensively studied organisms in nature, with the ultimate goal of obtaining a complete understanding of physiological processes that constitute the life of a cell. To this end, the genome, transcriptome and proteome. of *B. subtilis* have been and continue to be extensively investigated. The genome of *B. subtilis* was recently resequenced and reannotated [1] and comprises approximately 4200 genes [1]. In the earlier *Bacillus* functional analysis programme it was attempted to inactivate all unknown genes, which allowed the identification of around 270 essential genes and elucidated the functions of many gene products [2]. Transcriptome analyses have made it possible to study genome-wide responses of microorganisms to changes in their environment. In this manner, many transcriptional regulators and the members of their regulons have been identified in *B. subtilis*. Recently, all the transcriptionally active regions of the genome of *B. subtilis* were identified using tiling arrays. This study measured genome-wide expression of *B. subtilis* genes in rich (LB) medium and defined minimal (M9) medium, using a single time point in the mid-exponential growth phase [3].

Numerous proteomics studies have been performed and major progress has been achieved towards the goal of identifying the entire proteome of *B. subtilis* [4] [5]. Many of these proteomics studies addressed the question which proteins were expressed under growth and starvation conditions [6] [7] [8].

Such studies are typically performed on chemically defined media. However, in research as well as in industrial applications, *B. subtilis* is usually grown in rich medium. Growth on such media is an extreme example of mixed substrate utilization, since they are composed of complex carbon and nitrogen sources of undefined composition. By using DNA microarrays in a temporal way, the microorganism could be used as a live sensor to report on its gene expression changes. However, to date most transcriptome studies are not time-resolved, or contain fewer than 8 time points [9].

A large time-resolved transcriptome data set of *B. subtilis* has been lacking, although this can provide valuable information about the dynamic regulation of gene expression in this model organism. Therefore, in this study we performed a large time-resolved transcriptome analysis of *B. subtilis* with frequent sampling (40 time points). The purpose was to obtain a dynamic view of gene expression of *B. subtilis* during the entire growth curve on rich medium. In order to make our data as useful as possible for the scientific community, we have used an aerated batch culture as experimental setup to mimic the laboratory conditions that most researchers work with.

Using this approach, a detailed view of transcription of the entire genome of *B. subtilis* during exponential, transition and stationary growth phases in rich medium was obtained. On the basis of gene expression profiles, the stationary phase can be clearly divided into an early stationary phase and a late stationary phase. This study indicates the occurrence of stress responses during the transitions between different growth phases. Of these, the one due to nutrient limiation at the end of the exponential growth phase was expected, but also a remarkably large response of the SigW regulon occured precisely at the transition between the early and the late stationary phase. Since these stress responses were not induced by external stimuli, our results shed more light on the natural occurrence of stress responses during growth and their physiological function.

The data were analyzed with a number of in-house developed bioinformatics tools in order to functionally characterize the dataset and identify novel regulatory motifs. In conclusion, the large transcriptome data set forms a rich repository for the microbial scientific community, where the expression profile of each gene can be found during the entire growth curve of *B. subtilis* on commonly used rich medium.

## Results

### Growth curve

**Figure 1:**
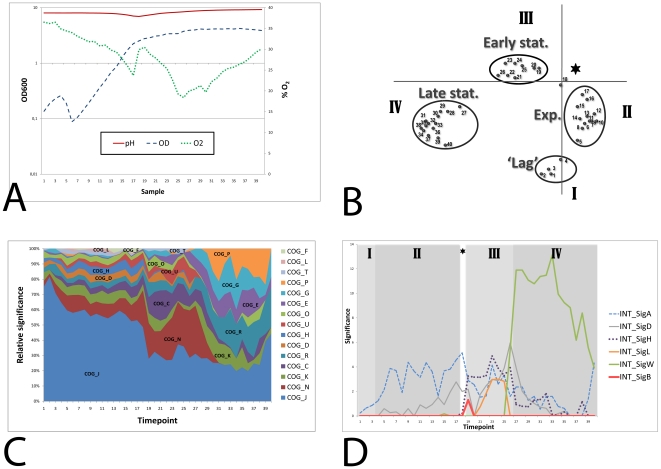
Growth curve and general characterization of the time-series experiment. A) Optical density (OD_600_), pH and oxygen levels (% O2) of the culture were monitored. B) Visualization of the first two components from the PCA analysis. I: lag phase, II: exponential growth phase, III: early stationary phase, IV: late stationary phase. The asterisk represents the transition point. C) Significantly overrepresented COG classes (log transformed p-values (á = 0.05 and Bonferroni corrected)) scaled per time point. COG_J: Translation, ribosomal structure and biogenesis, COG_K: Transcription, COG_L: DNA replication, recombination and repair, COG_D: Cell division and chromosome partitioning, COG_O: Posttranslational modification, protein turnover, chaperones, COG_N: Cell motility and secretion, COG_P: Inorganic ion transport and metabolism, COG_T: Signal transduction mechanisms, COG_C: Energy production and conversion, COG_G: Carbohydrate transport and metabolism, COG_E: Amino acid transport and metabolism, COG_F: Nucleotide transport and metabolism, COG_H: Coenzyme metabolism, COG_U: Intracellular trafficking and secretion, COG_R: General function prediction only. D) Significantly overrepresented sigma factor regulons (log transformed p-values (á = 0.05 and Bonferroni corrected)). The different growth-phases that were identified by the PCA analysis are visualized in the expression graph. I: lag phase, II: exponential growth phase, III: early stationary growth phase. IV: late stationary growth phase. The asterisk represents the transition point.

In this study, we set out to obtain a highly detailed view of the temporal transcriptome changes of *Bacillus subtilis* grown under commonly used laboratory conditions. For this purpose, *B. subtilis* 168 was grown in an aerobic batch culture in TY (trypton/yeast extract) medium at 37°C. The pH and oxygen concentrations were monitored, but not controlled. The culture was inoculated at an OD_600_ of 0.01, and exponential growth was shown to start soon after. When the OD_600_ reached 0.1, samples were withdrawn every 10 minutes for 40 time points. Growth of the culture was monitored by measurement of the OD_600_ (Figure 1A) and could be divided in four distinct growth phases. Directly after inoculation the OD_600_ of the culture increased exponentially, but after time point 4 the OD suddenly decreased severely, from 0.26 to 0.09 within 20 minutes. After this, from time point 6 onwards, the culture again grew exponentially. This brief drop in OD was consistently observed in 3 similar culture fermentations around OD_600_ = 0.3, although the severity of the OD decrease and the exact timing of this event slightly differed (data not shown). We have termed the first 4 time points the ‘lag’ phase. The possible cause of this decrease during lag phase is unknown and it cannot be excluded that it depends on our method of inoculation with preculture. The second growth phase, the exponential phase, continued until time point 18, when a distinct transition point was reached at an OD_600_ of 2.25. In the third phase, the early stationary growth phase, the OD_600_ slowly increased, while it decreased slightly in the late stationary phase, phase four. Although the aeration of the culture was kept constant, the oxygen level fluctuated during the growth curve (Figure 1A). During exponential growth, the oxygen level continuously decreased. After a short increase at the transition point, the level again decreased during the early stationary phase until a minimum was reached and the oxygen level increased again during the late stationary phase. The pH of the culture gradually decreased from around pH 8 to pH 7 at the transition point, after which it steadily increased again to above pH 9 at the end of the experiment. These fluctuations in oxygen level and pH were reproducible in repeated experiments (data not shown).

### Principal Component Analysis

RNA was isolated from these 40 samples and microarray analyses were performed and data were analyzed with established methods. We first examined the data with a Principal Component Analysis (PCA). This method is used for dimension reduction of complex data sets, by transforming a number of possibly correlated variables into uncorrelated variables called principal components of which a selected number is considered. The first component represents the highest variability in the data, and each succeeding component represents a portion of the remainder of the variability. The first two components are visualized in a graph (Figure 1B) in which distinct clusters of time points can be clearly observed. These clusters correspond to the growth phases that we identified from the growth curve. Thus we can conclude that the transitions between the growth phases are reflected in the expression profiles of the genes. According to this PCA, the lag phase consists of time points 1 to 4, whereas time points 5 and 6 rather belong to the exponential phase. There is a very clear transition point: time point 18. Remarkably, the early and late stationary phases are not continuous, but are clearly separated.

### Functional enrichment analysis

Genes were grouped into functional categories and analyzed by the iGA algorithm [10] from the FIVA application [11]. Functional categories were based on metabolic pathways from KEGG [12], categories from Gene Ontology [13] and regulons from DBTBS [14]. The iGA algorithm determines the optimal expression threshold for each timepoint for each functional category. This approach allows for the identification of overrepresented categories which are moderately induced. This analysis indicates which processes occur prominently at particular moments in the growth curve. In Figure 1C, the significantly overrepresented COG categories are shown. The most active process during growth and starvation is translation, since the category COG J (translation) is most significantly overrepresented during the entire growth curve. Several COG categories become more active after the transition point, most notably COG N (Cell motility and secretion) and COG C (Energy production and conversion). During the late stationary growth phase, the categories COG P (Inorganic ion transport and metabolism), COG G (Carbohydrate transport and metabolism), COG E (Amino acid transport and metabolism), COG K (Transcription) and COG R (General function prediction only) are most prominently overrepresented.

### Response to oxygen

To investigate genes that respond to the varying concentrations of oxygen or cause the oxygen concentration to change, we have performed a correlation analysis using the oxygen percentages and expression levels of the genes (see material & methods). The top correlating (i.e., genes that display an expression profile that is similar) and anti-correlating genes (i.e., genes that display an expression that is opposite) with the changing oxygen percentage are described in Table 1. Some of these genes have indeed already been implicated to be influenced by oxygen (see Discussion).

**Table 1:** Genes with expression profiles correlating with oxygen concentration.

Gene Name	Positive Correlation	Literature reference	Gene Name	Negative Correlation	Literature reference
*yrkD*	0.84	n.a.	*ybdO*	−0.90	n.a.
*ykyA*	0.82	n.a.	*ylqB*	−0.89	n.a.
*yacN (ispF)*	0.81	[29]	*sdpC*	−0.88	[32]
*yqiG*	0.81	[28]	*rapH*	−0.88	n.a.
*ydhL (pbuE)*	0.81	n.a.	*smf (dprA)*	−0.87	n.a.
*yvsG*	0.80	n.a.	*yqaP*	−0.87	n.a.
*yveK (epsA)*	0.79	[31], [30]	*ydcM (immA)*	−0.85	n.a.
*yckA*	0.78	[30]	*yydG*	−0.85	n.a.
*yktD*	0.77	n.a	*yokG*	−0.85	n.a.

The genes with expression profiles most correlating and anti-correlating with the oxygen concentration. Some of these genes have already been implicated with oxygen or related phenomena. (n.a.: not available).

#### Transition point

The transition phase is defined as the growth phase where a culture ceases exponential growth and enters the stationary growth phase. The Principal Component Analysis of our data set suggested that the transition phase in our experiment consists of a single timepoint. Most likely this represents the phase where the cells switch their metabolism from growth on glycolytic carbon sources to gluconeogenesis. Therefore the timing of this switch was investigated by looking at the expression profiles of *gapA* and *gapB*. These genes encode the two glyceraldehyde 3-phosphate dehydrogenases of *B. subtilis*, of which GapA is expressed during glycolytic growth (mediated by the transcriptional repressor CggR), and GapB is expressed under gluconeogenic conditions. As expected, the expression profiles of these genes in our time series indicate a clear and rapid switch from glycolysis to gluconeogenesis at the transition point (see **Figure S1A**). In addition, the expression profiles of the genes involved in acetate production and consumption were analyzed. Acetyl CoA is converted into acetate by the products of *pta* and *ackA*, while the enzyme AcsA catalyzes the reverse reaction. The expression profiles of these genes show a clear and very rapid switch between the production and utilization of acetate at the transition point (see **Figure S1B**). Another class of genes that showed a response during the transition point comprises of phage related genes. The category containing these genes was found overrepresented for timepoints 16–19 (see **Figure S2**).

### Sigma Factors

*Bacillus subtilis* employs different sigma factors under different environmental conditions to regulate a wide range of physiological processes. We analyzed which regulons consisting of genes that are controlled by one of the alternative sigma factors were significantly expressed during our experiment. Figure 1D shows the activity of the sigma factor regulons of *B. subtilis* that are significantly overrepresented during any part of the growth curve. As expected, the general sigma factor of *B. subtilis*, SigA, is the primary sigma factor, since the SigA regulon is the most significantly overrepresented regulon during the exponential growth phase. The SigB regulon, which is induced by various stresses and upon entry into stationary growth, is only briefly overrepresented at time point 19, indicating that a slight natural stress response occurs at the transition point. SigD is the sigma factor responsible for flagella synthesis and chemotaxis and its regulon is quite active, mainly in the early stationary phase. SigH is the sigma factor involved in early stationary phase processes, including the initiation of sporulation. The SigH regulon is indeed active in the early stationary phase, but categories related to sporulation were never overrepresented during our experiment (not shown). The regulon of SigL, the sigma factor that regulates catabolism of some amino acids as well as acetoin and levanan degradation, is only active in the early stationary phase. Finally, the extracytoplasmic sigma factor SigW regulon shows a large response that marks the transition from the early to the late stationary growth phase. This sigma factor is known to be induced by membrane and cell wall stress caused by antimicrobial compounds produced by other Bacilli [15]. In this experiment, we show that it is also naturally induced during growth in rich medium. Some possible causes are investigated below.

### Sporulation delay protein (Sdp) and sporulation killing factor (Skf)

Nutrient limitation triggers spore formation, which is accompanied by the induction of the *sdp* (sporulation delay protein) and *skf* (sporulation killing factor) operons. These toxic peptides serve to lyse nonsporulating sibling cells in a process termed cannibalism [16] [17] [18] [19]. We investigated the expression profiles of these genes to analyze whether they could be responsible for the observed induction of the SigW regulon.

**Figure 2:**
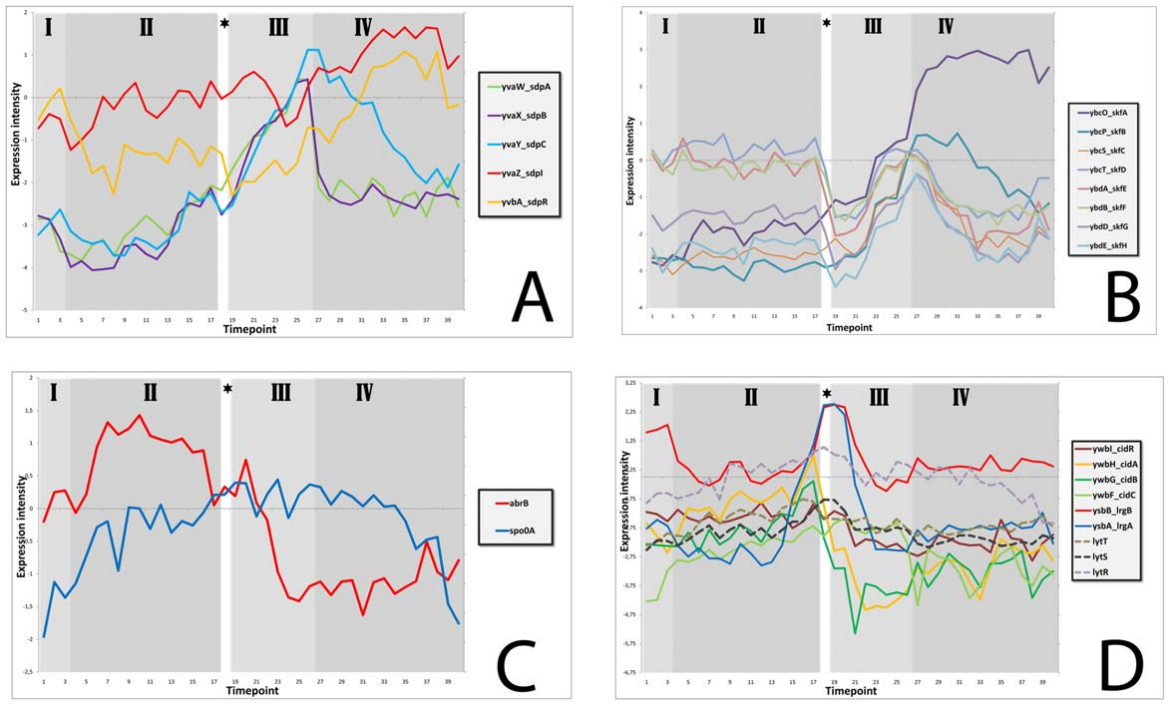
Expression profiles of genes involved in the production of toxic proteins. A) The *sdp* operon. B) The *skf* operon. C) The regulators AbrB and Spo0A. D) Antiholin *lrgAB*, holin *cidA*, remaining members of operon *ywbGF*, putative regulator *ywbI*, and the *lytST* two-component regulatory system.

The expression of the *sdpABC genes* was clearly activated in the early stationary phase and decreased at the onset of the late stationary phase. In contrast, the expression of the immunity genes *sdpI* and s*dpR* remained relatively constant and continued into the late stationary phase (see Figure 2A).

The expression profile of the *skf* operon shows that it is also activated at the early stationary phase (Figure 2B). The *skfA* gene, which encodes the killing factor itself, continues to be highly expressed during the late stationary phase, whereas expression of the other genes in the operon decreases.

The activation of the *sdp* and *skf* operons could be the reason for the observed SigW response. Although the SdpI protein provides the primary immunity to SdpC, the SigW regulon gives important secondary assistance, especially when cells are grown in liquid medium [15] [20]. The SigW-dependent SdpC resistance is caused by *yknWXYZ*, encoding an ABC transporter and to a lesser extent by *yfhLM* [15]. Both operons are clearly activated at the onset of late stationary phase (see **Figure S3**). Whereas expression of *yknWXYZ* decreases as soon as the *sdp* operon is less active, the *yfhLM* operon remains activated.

Both *skf* and *sdp* operons are activated by a low concentration of Spo0A∼P, which relieves AbrB repression of these genes [21]. The expression of Spo0A remains relatively constant during growth, but the amount of the active, phosphorylated form cannot be distinguished in these DNA microarrays. The expression of the transition state regulator AbrB decreases at the end of the exponential phase and reaches its lowest levels at the onset of the late stationary phase (See Figure 2C).

### Programmed Cell Death

Programmed Cell Death has been mainly investigated in *S. aureus*, but this process seems to be conserved among gram positive bacteria and *B. subtilis* contains homologues of the genes involved in this phenomenon [22] [23] [24]. In *B. subtilis*, CidA (*ywbH*) presumably functions as a holin, LrgA (*ysbA*) as the corresponding anti-holin, and YwbI as a putative transcriptional regulator, whereas the functions of YwbG, YwbF and LrgB (YsbB) are currently unknown. A slight induction of the *cidA-ywbGF* operon and a much higher induction of the *lrgAB* operon can be observed around the transition point (see Figure 2D). In *S. aureus*, *lrgAB* transcription is positively regulated by the LytSR two-component regulatory system encoded immediately upstream which senses a decrease in membrane potential [22]. In *B. subtilis* the genomic organisation of these genes is similar, with the LytS-LytT two-component system encoded upstream of the genes *ysbA* and *ysbB*. Therefore, it seems likely that regulation works similarly, but since two-component signal transduction occurs via protein phosphorylation, large changes in the expression levels of LytST are not observed (See Figure 2D).

#### Whole time-series analysis by bioinformatics

Instead of only looking at expression profiles of individual genes or operons, we have developed new bioinformatic tools in order to extract information from time series transcriptome data by comparing expression profiles. The DISCLOSE [25] software combines clustering with functional information and can identify putative regulatory motifs within clusters.

### Clustering analysis and most overrepresented motifs

We developed an exploratory application, coined DISCLOSE [25], that benchmarks the results of clustering methods using a DNA motif discovery algorithm and functional annotations. The motif discovery algorithm identifies overrepresented DNA sequences, which could be indicative of sites of transcriptional control, in the upstream DNA sequences of genes from the clusters. The functional annotations are used to characterize these motifs by identifying significantly enriched categories using the genes that contain the motif. The software allows researchers to distinguish between known and unknown binding sites by automatically comparing identified motifs with known DNA binding motifs of regulators. In addition to the comparison with known motifs, the DISCLOSE software also provides a graphical representation of the genomic context of the putative and known motifs in the upstream regions of the operons. This visualization indicates the location of the putative motif (closed boxes) and also displays known motifs (open boxes). The graphical representation of the genomic context of putative and known DNA binding sites greatly facilitates the identification of known DNA binding sites.

The manual clustering analysis of our time series transcriptome was performed using a large number of clusters. This resulted in small clusters, several of which display a very specific expression behavior. A total of 13 binding sites were discovered by our DISCLOSE application that overlapped with known DNA binding sites (see **Figure S4**). One of the identified binding sites that was similar to a known binding site was not yet catalogued in the transcription factor database, but was already described in the literature. The gene *ycgT*, that did not contain a known Fur binding site at the time of the analysis, was recently added to the DBTBS database as member of the Fur regulon. Other targets with binding sites similar to a known binding site have been postulated in the literature, but are still not tested experimentally. For example, DISCLOSE identified a motif in the upstream region of *pucA* that is similar to the known binding site of TnrA. A different study already speculated that *pucA* might be induced by TnrA under nitrogen-limiting conditions [26].

**Figure 3:**
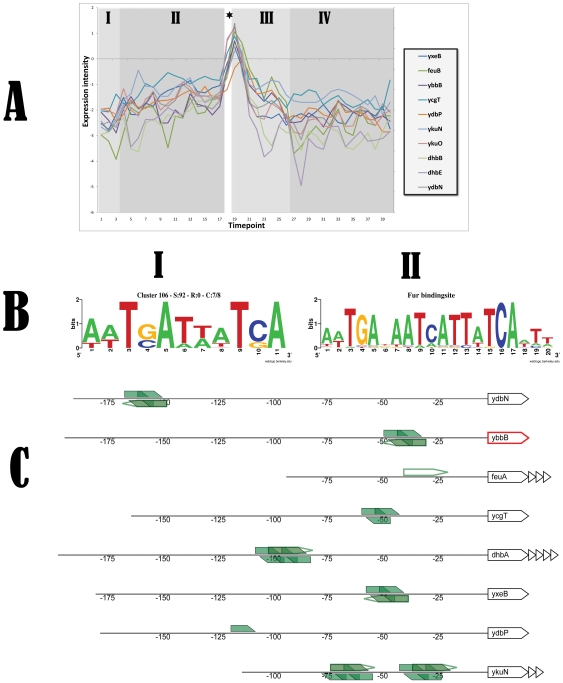
Information for cluster 106 obtained by a K-means clustering analysis using 189 clusters. The most overrepresented DNA binding site was identified in the upstream regions of the operon members of this cluster. A) Expression graph of the members of cluster 106. B.I) Most overrepresented motif that was identified in 7 out of 8 operons of the cluster. B.II) The most overrepresented motif from B.I shows high similarity with the known DNA binding site of Fur. C) Visualization of the genomic context of putative and known motifs in the upstream sequences of the operons. Open polygons represent known binding sites derived from literature sources. Filled polygons depict putative motifs, numbers represent the upstream region. The first gene of the operon is displayed; remaining members of this operon are visualized as triangles. Genes coding for a putative regulator are colored red.

The most overrepresented motif was found in a cluster that comprised 10 genes (corresponding to 8 operons) and was induced upon entry in the stationary phase (see Figure 3). Our analysis revealed that the identified motif was highly similar to the known DNA binding site of the Fur regulator of *B. subtilis*. In this case, the found motif overlaps with the known Fur motif. All members of this cluster containing this putative motif are already annotated as members of the Fur-regulon.

Several motifs that were examined during our analysis were marked ‘putatively interesting’ based on their occurrence pattern or other typical motif features. Some examples of these motifs are listed in Figure S5. Both genes (*trkA* and *czcD*) from cluster 124 are involved in the uptake of potassium, which could be indicative of the biological function related to the identified motif. In addition, the motif that was found in four members of cluster 146 overlaps with a previously identified inverted repeat that may represent a YfmP binding site [27]. Many other putative motifs were discovered, and the results of our complete motif analysis can be found at the **supplemental website**. These putative motifs might be very helpful for choosing future research directions.

## Discussion

In this study we have determined the transcriptome of *B. subtilis* during lag, exponential and stationary growth phases and at the transition point on rich medium by sampling every 10 minutes. By examining the expression levels for the genes at each time point, we were able to distinguish distinct growth phases and a very clear transition point. The high number of sampling points that was used for our experiment has allowed various powerful bioinformatic analyses.

A correlation analysis that considered the varying concentration of oxygen as expression value identified several interesting genes whose expression levels correlated or anti-correlated with the oxygen concentration. A positively correlating gene was *yqiG*, which was described in a study of Mostertz et al. These authors characterized the peroxide and the superoxide stress stimulons of *B. subtilis* by means of transcriptomics and proteomics [28]. Amongst others, they found that *yqiG* exhibited increased expression after H_2_O_2_ and after paraquat challenge. Also the expression of *yacN* (*ispF*) has previously been found to be influenced by stress stimuli that increase the level of reactive oxygen species [29]. A very recent article describes a transcriptome analysis of *B. subtilis* during fermentation with low or high dissolved oxygen concentrations [30]. In this study transcription of *yveK* (*epsA*) as well as *yckA* was shown to be repressed at the low oxygen concentration. This confirms our finding that the expression profiles of these genes positively correlates with the oxygen concentration. *EpsA* is part of the *epsA-O* operon involved in exopolysaccharide production during biofilm formation. An investigation of biofilm formation of *B. subtilis* on the surface of plant roots seems to contradict the positive correlation with oxygen [31]. In this study the *epsA-O* operon (and biofilm formation) was downregulated by cathechol, an effect mediated by radical oxygen species. This contradicting result might be explained by the different experimental setup, since the downregulation was observed under biofilm formation conditions and occurred only in the late stationary phase. Interestingly, the gene with the third best anti-correlation with oxygen was *sdpC* (formerly known as *yvaY*), sporulation delay protein. It encodes a toxic peptide that serves to lyse nonsporulating sibling cells in a process termed cannibalism (discussed in more detail below) [17]. The *sdp* operon was previously found to be induced under anaerobic conditions [32], which is confirmed by our data (see **Figure S6** for the expression profiles of *sdpC* and *yqiG* and the oxygen levels).

In addition, clustering approaches were examined using a motif identification algorithm, using the in-house developed application DISCLOSE [25] in order to functionally characterize the dataset and identify novel regulatory motifs.

The motif discovery algorithm identified 13 overrepresented DNA sequences which were found to overlap with known DNA binding sites of transcriptional regulators (see **Figure S4**). One of these, identified in the promoter region of the gene *ycgT*, was determined by DISCLOSE to overlap with the Fur recognition sequence. This result was recently confirmed when this gene was added to the DBTBS database as a member of the Fur regulon. In addition, a motif in the upstream region of *pucA* similar to the known binding site of TnrA was identified by DISCLOSE. This is supported by an earlier study that already speculated that *pucA* might be induced by TnrA under nitrogen-limiting conditions [26]. The most overrepresented motif was found in a cluster that comprised 8 operons and was induced upon entry in the stationary phase (see Figure 3). The analysis revealed that the identified motif was highly similar to the known DNA binding site of the Fur regulator of *B. subtilis*. Indeed, all members of this cluster were already annotated as members of the Fur-regulon. Taken together, these results convincingly demonstrate that the DISCLOSE application can identify transcription factor binding sites. Therefore, we believe that the other motifs we identified with DISCLOSE (which can be found at the **supplemental website**) are putative transcription factor binding sites that are promising targets for further research. Some examples are depicted in Figure S5. For example, cluster 124 consists of two operons (3 genes in total), which are both described to be involved in the uptake of potassium. This therefore might be indicative of the biological function related to the motif that was identified in the upstream regions of these operons. In addition, the motif that was found in four members of cluster 146 overlaps with a previously identified inverted repeat that may represent a YfmP binding site [27]. One of the upstream regions where this motif was found was that of *yfmP* itself, suggesting that the gene is subject to auto-regulation.

Among the most notable findings of our analyses was the identification of several stress responses that occurred without applying external stresses. The first symptom of stress that *B. subtilis* showed in our experiment is a small but significant induction of the SigB regulon at the transition point, which is most likely caused by energy stress. In addition, phage related genes were also activated during the transition point. A much stronger response is observed for the SigW regulon, of which most members are highly upregulated during late stationary phase. It is known that this regulon provides resistance against antimicrobial compounds, including SdpC [15]. The expression profiles of the genes of the *sdp* and *skf* operons show that they are activated during the stationary growth phase (Figure 2A and Figure 2B) and therefore could be at least partly responsible for the induction of the SigW regulon. In the *sdp* operon, the exported toxic protein SdpC mediates killing. SdpI (*yvaZ*), the immunity protein, responds to the SdpC toxin by sequestering the SdpR (*yvbA*) autorepressor at the membrane. This sequestration relieves repression of the *sdpRI* operon and thus stimulates synthesis of the immunity protein [33]. In our experiment, the expression of *sdpI* and *sdpR* continued into the late stationary phase, whereas expression of the *sdpABC* genes decreased (Figure 2A), as has been observed before [8]. In the *skf* operon, the presence of a potential stem-loop structure between *skfA* and *skfB* accounts for the high stability of the *skfA* (*ybcO*) transcript (see Figure 2B) which encodes the killing factor itself [34].

Both *skf* and *sdp*, as well as *sigW*, are negatively regulated by AbrB [21] [35]. This repressor is present during exponential growth, but nutrient depletion and subsequent activation of Spo0A leads to a decrease in *abrB* expression when cells enter the stationary phase which relieves AbrB repression. Our results indeed show that *abrB* expression decreases at the end of the exponential phase and reaches its lowest levels at the onset of the late stationary phase (Figure 2C). Clearly, AbrB levels become low enough to allow activation first of the *sdp* and *skf* operons and later of the *sigW* regulon which can provide additional resistance to Sdp and Skf. These operons are also under control of other regulatory mechanisms via Spo0A , Abh, AbbA, and (in the case of SigW) cell wall stress [21] [35] [36] [37], which may explain the difference in timing of activation. For example, *skf* is also directly activated by low levels of Spo0A∼P, while the *sdp* operon is repressed by a high concentration of Spo0A∼P. This might explain why Sdp is only transiently activated, whereas Skf continues to be produced as the concentration of Spo0A∼P rises [21].

Genes which are possibly involved in Programmed Cell Death in *B. subtilis* [22] [23] are only slightly induced around the transition point (see Figure 2D), with the putative holin CidA (*ywbH*) being expressed to a lesser extent than the putative anti-holin LrgA (*ysbA*). Perhaps this indicates that the holin CidA is only induced in a subpopulation of cells, causing them to lyse. Release of the holin in the medium might then result in induction of the antiholin LrgA in the rest of the culture. By analogy to the process in *S. aureus*, the LytST two-component regulatory system would sense a decrease in membrane potential and subsequently induce *lrgAB* transcription. The expression levels of LytST do not change, which is understandable since signal transduction would occur via protein phosphorylation (See Figure 2D). This system might contribute to the induction of the SigW regulon, but since little is known about the function of any these proteins in *B. subtilis* yet, we cannot draw a more definite conclusion.

In conclusion, *B. subtilis* experiences stresses during its growth curve without researchers imposing them. The DNA microarray data generated by this time series experiment is very useful for mining with bioinformatics tools, allowing for example the identification of novel putative gene functions and regulatory motifs. We therefore expect our data to be a rich repository for the scientific community (both microbiologists and bioinformaticians) in which the dynamic expression profile of each gene of *B. subtilis* can be found during the entire growth curve on rich medium. The clear and distinct growth phases in this data set can also be used in the transcriptome analysis of existing and future perturbation studies (e.g., gene knock-outs, overexpression, changing growth conditions) to filter out genes that are affected by changes in growth phase.

## Materials and Methods

### Bacterial strain and culture conditions

Wild-type *Bacillus subtilis* 168 (*trpC2*) was grown in TY medium (10 g/L trypton, 5 g/L yeast extract, 5 g/L NaCl) in batch mode at 37°C in a 16 L fermentor using a working volume of 12 L. Cultures were aerated at 40 L/min and 400 rpm. The pH and oxygen levels were monitored, but were allowed to fluctuate during the batch culture incubation period. To prevent foaming, small aliquots of 0.3% antifoam A solution (SIGMA) were added on an as-needed basis. Cultures were inoculated to an OD_600_ of approximately 0.01 from synchronized exponentially grown pre-cultures grown at 37°C in TY and frozen in 10% glycerol [38], which resulted in an initial concentration of glycerol of 0.08%. Growth was monitored by measurement of the OD at 600 nm. As soon as the culture reached an OD_600_ of 0.1 (typically after around 75 min.), 50–200 ml samples were withdrawn every 10 minutes for optical density measurements and RNA isolation. Earlier sampling was not feasible in view of the large volumes (a total of 3.2 L of medium was retrieved for RNA isolation) needed. In total 40 time points were sampled. Cells were harvested by centrifugation (14.000 rpm, 4°C, 1 min.) and the pellet was immediately frozen in liquid nitrogen and stored at −80°C. The OD measurements for this culturing method were performed in triplicate cultures and gave very similar growth curves each time.

### RNA isolation and cDNA generation

RNA was isolated and DNA microarray analyses were performed essentially as described [38]. RNA was isolated with the High Pure RNA Isolation Kit (Roche Applied Science) according to the manufacturer's instructions. RNA quantity and quality were assessed with a Nano Drop ND-1000 spectrophotometer (NanoDrop Technologies) and an Agilent Bioanalyzer 2100 with RNA 6000 LabChips (Agilent Technologies Netherlands BV). cDNA was synthesized with the Superscript III Reverse Transcriptase kit (Invitrogen) using 10 µg of total RNA as template and 360 U of Superscript™ III RT and purified with the Cyscribe GFX purification kit (Amersham Biosciences). The purified cDNA was labeled with Cy3- or Cy5-monoreactive dye and purified again with the Cyscribe GFX purification kit. The labeled cDNA was hybridized to oligonucleotide microarrays [38] in Ambion Slidehyb #1 buffer (Ambion Europe Ltd) at 45°C for 16–18 h. After hybridization, slides were washed for 5 min at 37°C in 2× SSC with 0.5% SDS, 2×5 min at 37°C in 1× SSC with 0.25% SDS and 1 min at 37°C in 1× SSC with 0.1% SDS, and then dried by centrifugation (2 min, 2000 rpm). SSC = 150 mM NaCl, 15 mM trisodium citrate.

### DNA microarray experiment design

For each time point, labeled cDNA was hybridized to a microarray slide together with labeled cDNA from the previous time point. In addition, the same DNA was used in a dye swap hybridization with the labeled cDNA from the subsequent time point. Moreover, to ensure that every time point was sampled three times, labeled cDNA from each time point was hybridized with labeled cDNA from 30 mins later (e.g., 1 vs 4, 2 vs 5 etc.). The three slides resulted in six measurements per gene, since each slide contains two duplicate spots for all genes.

### DNA microarray analysis

The microarrays were scanned with a GenePix Autoloader 4200AL confocal laser scanner (Molecular Devices Ltd). Determination of the individual intensities of each spot was done with ArrayPro 4.5 (Media Cybernetics Inc., Silver Spring, Md., USA) with a local corners background correction method and the resulting expression levels were processed and normalized (Lowess method) with MicroPrep [39]. The median expression level was calculated for all genes based on the expression values from all six intensity measurements for each time point. To facilitate the analysis of expression levels of genes for each time point we divided the median expression level for each gene by the averaged expression values of all genes for each time point, which was followed by a natural log transformation. The resulting normalized intensity data for each individual time point were used for subsequent analyses. The normalized data matrix for all time points as well as the raw microarray data from this study have been submitted to the NCBI Gene Expression Omnibus (http://www.ncbi.nlm.nih.gov/geo) under accession no. GSE19831 in a format that complies with MIAME guidelines.

A Principal Component Analysis (PCA) was applied to our dataset using the TM4 software package (http://www.tm4.org) to examine the internal variation in expression levels of all genes for each time point. Additional applications were employed for the downstream analysis of the expression data, these applications are described below.

### Functional enrichment analysis

The FIVA [11] software was used to perform the functional enrichment analysis on genes from the most expressed fractions at each of the time points. Various annotation sources were used in this enrichment analysis: terms from the controlled vocabulary Gene Ontology [13], metabolic pathways from KEGG [40], InterPro motifs [41], keywords from UniProt [42] and regulons from the DBTBS database [14]. Several multiple testing corrections were employed to correct the raw *p*-values obtained from the analyses [11].

#### Oxygen concentration correlation analysis

A correlation analysis was performed using the oxygen percentages. For this purpose, we created an artificial gene and considered the oxygen concentrations at each time point as expression values for this hypothetical gene. Next, for all genes and the artificial oxygen gene, the expression profiles were used to create a correlation matrix using the Pearson correlation measure. Each row of this matrix represents the correlation between a gene and all other genes.

### Clustering analysis

The DISCLOSE software [25] was employed for a clustering analysis of the expression profiles from the 40 time points using the K-means algorithm for 100 to 200 clusters. In addition to the clustering, the DISCLOSE application was also used for the characterization of the clustering results using functional annotations. Moreover, a *de novo* DNA motif discovery algorithm [25] was used to identify overrepresented DNA motifs in the upstream sequences of genes from the clusters. Information concerning known DNA binding sites from the DBTBS database [14] was used for the visualization of the genomic context of the motifs. The results of a K-means clustering were analyzed for a range of clusters and correlation measures. Results for each clustering analysis were ranked based on the score of the most overrepresented motif. The best performing clustering run (K-means clustering for 189 clusters using uncentered Pearson correlation) was selected for a detailed analysis. In addition, the best performing clustering run based on the total number of motifs that exceeded a predefined threshold (threshold value of 20) was selected for further analysis (i.e., a K-means clustering for 197 clusters using Cosine correlation).

## Supporting Information

Figure S1 A) Expression profiles of the glyceraldehyde 3-phosphate dehydrogenases *gapA* and *gapB* and expression of the regulator *cggR*. B) Expression profiles of *pta*, *ackA*, *acsA* and *acoA*.
(TIF)

Figure S2 Significantly overrepresented category containing phage related genes (log transformed p-values (á = 0.05 and Bonferroni corrected)) scaled per time point. This category was found significantly overrepresented for timepoints 16–19.
(TIF)

Figure S3 Expression graph of *yknWXYZ* and *yfhLM* which mediate SigW dependent resistance to SdpC .
(TIF)

Figure S4 Overview of identified binding sites that overlapped with known binding sites. The first column displays the averaged expression level of the gene members of a cluster. The second column describes the name of the known regulator of which some known binding sites were identified. E.g.; the first cluster consists of 8 operon members of which 7 members contain the Fur binding site in their upstream regions that was identified by DISCLOSE. The identified motifs are visualized as sequence logos in the third column.
(TIF)

Figure S5 Overview of identified binding sites that do not match with known binding sites. The first column displays the averaged expression level of the gene members of a cluster. The second column describes the name of the cluster, the coverage of the motif and the first genes (genes representing putative or known regulators are indicated in bold) of the operons that contain the motif in their upstream region. E.g.; cluster 4 consists of 13 operons of which 3 members contain a putative binding site in their upstream regions that was identified by DISCLOSE. The identified motifs are visualized as sequence logos in the third column.
(TIF)

Figure S6 Examples of genes showing correlation (*yktD*) or anti-correlation (*sdpC*) with oxygen. The different growth-phases that were identified by the PCA analysis are visualized in the expression graph. I: lag phase, II: exponential growth phase, III: early stationary growth phase. IV: late stationary growth phase. The asterisk represents the transition point.
(TIF)
