# Supplementary figures and images for: Time-Resolved Transcriptomics and Bioinformatic Analyses Reveal Intrinsic Stress Responses during Batch Culture of Bacillus subtilis
Source: PLoS One. 2011 Nov 8;6(11):e27160. doi: 10.1371/journal.pone.0027160 (PMC3210768; doi:10.1371/journal.pone.0027160)

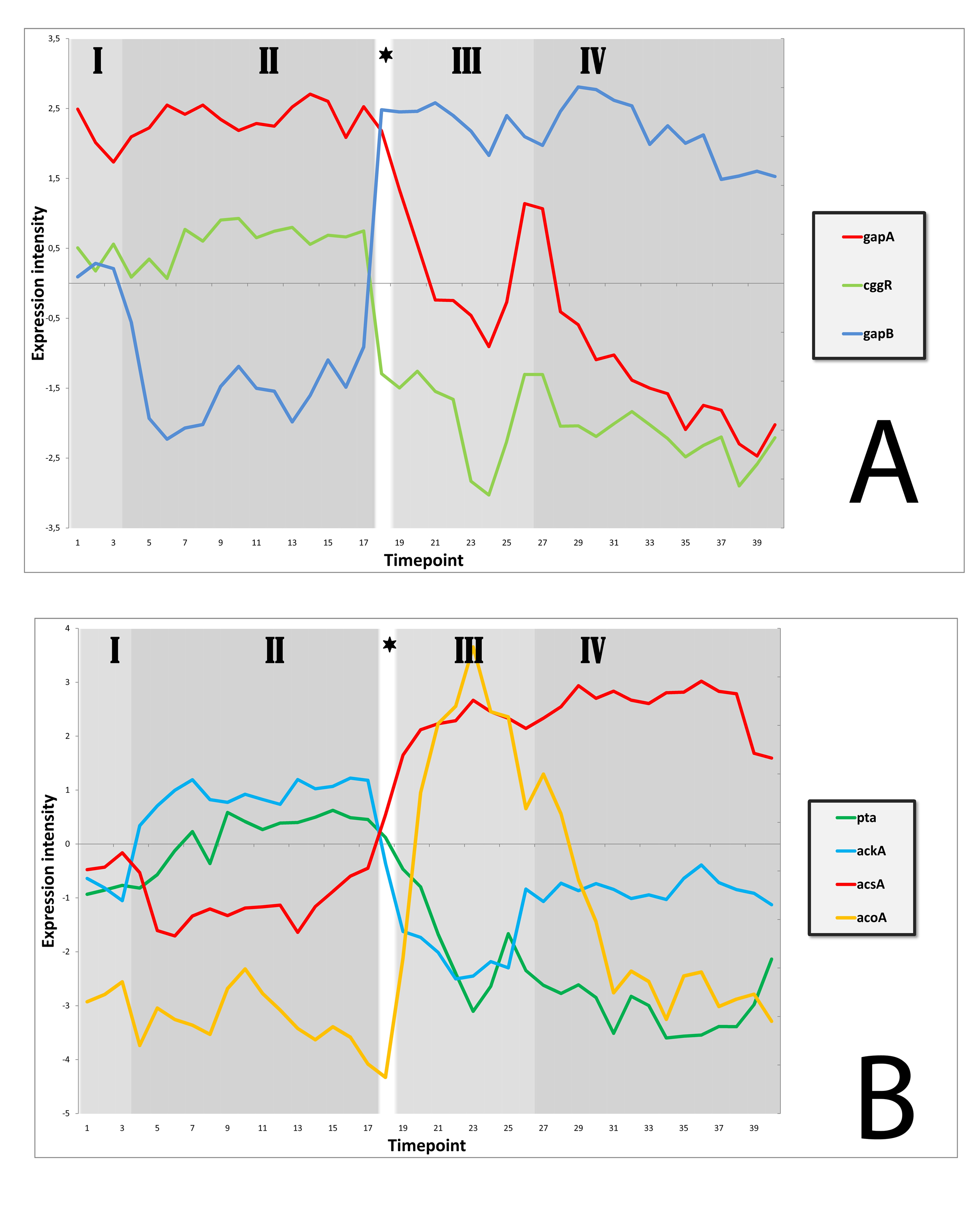

Supplement: Figure S1 — A) Expression profiles of the glyceraldehyde 3-phosphate dehydrogenases gapA and gapB and expression of the regulator cggR. B) Expression profiles of pta, ackA, acsA and acoA. (TIF) [file pone.0027160.s001.tif]

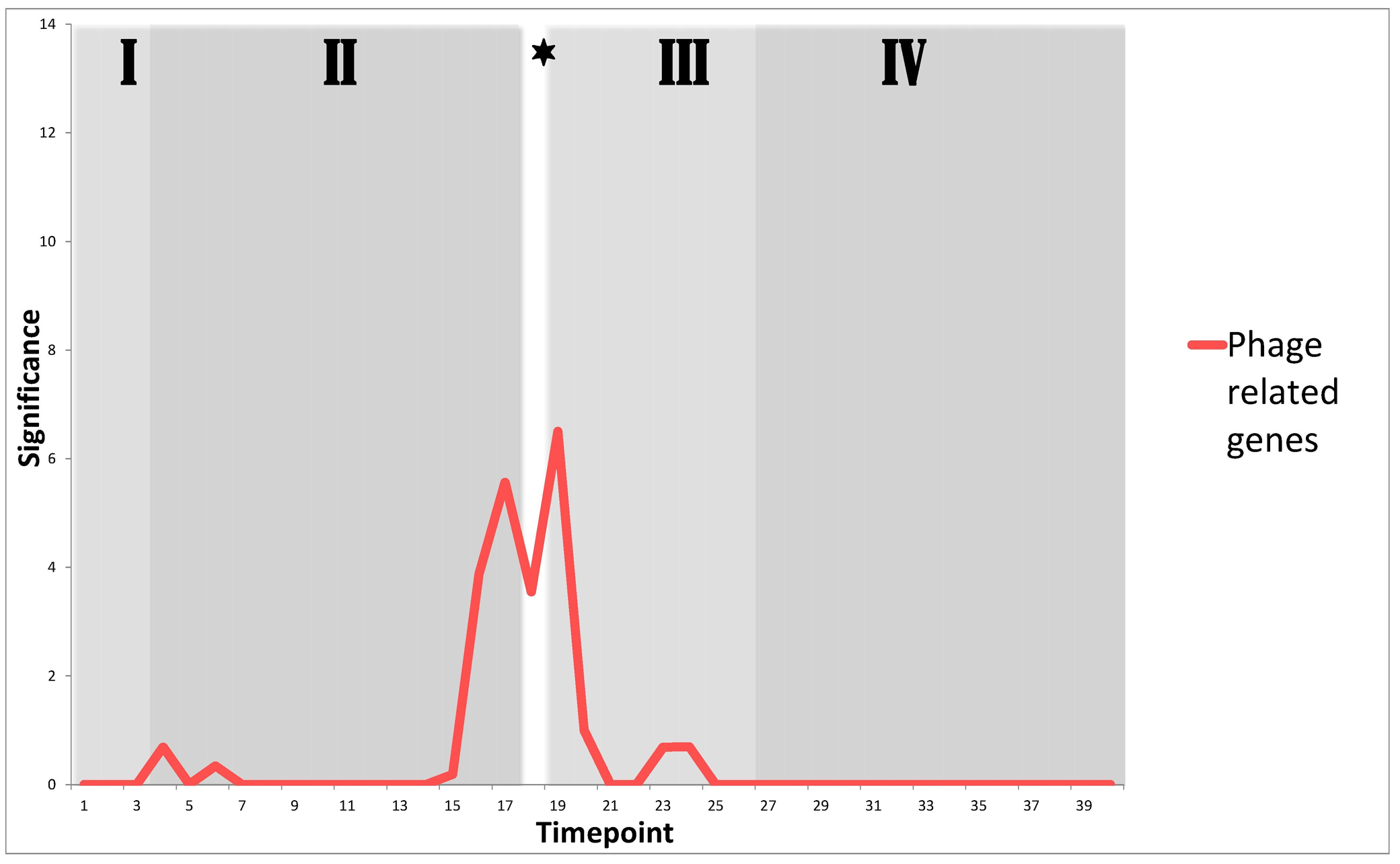

Supplement: Figure S2 — Significantly overrepresented category containing phage related genes (log transformed p-values (á = 0.05 and Bonferroni corrected)) scaled per time point. This category was found significantly overrepresented for timepoints 16–19. (TIF) [file pone.0027160.s002.tif]

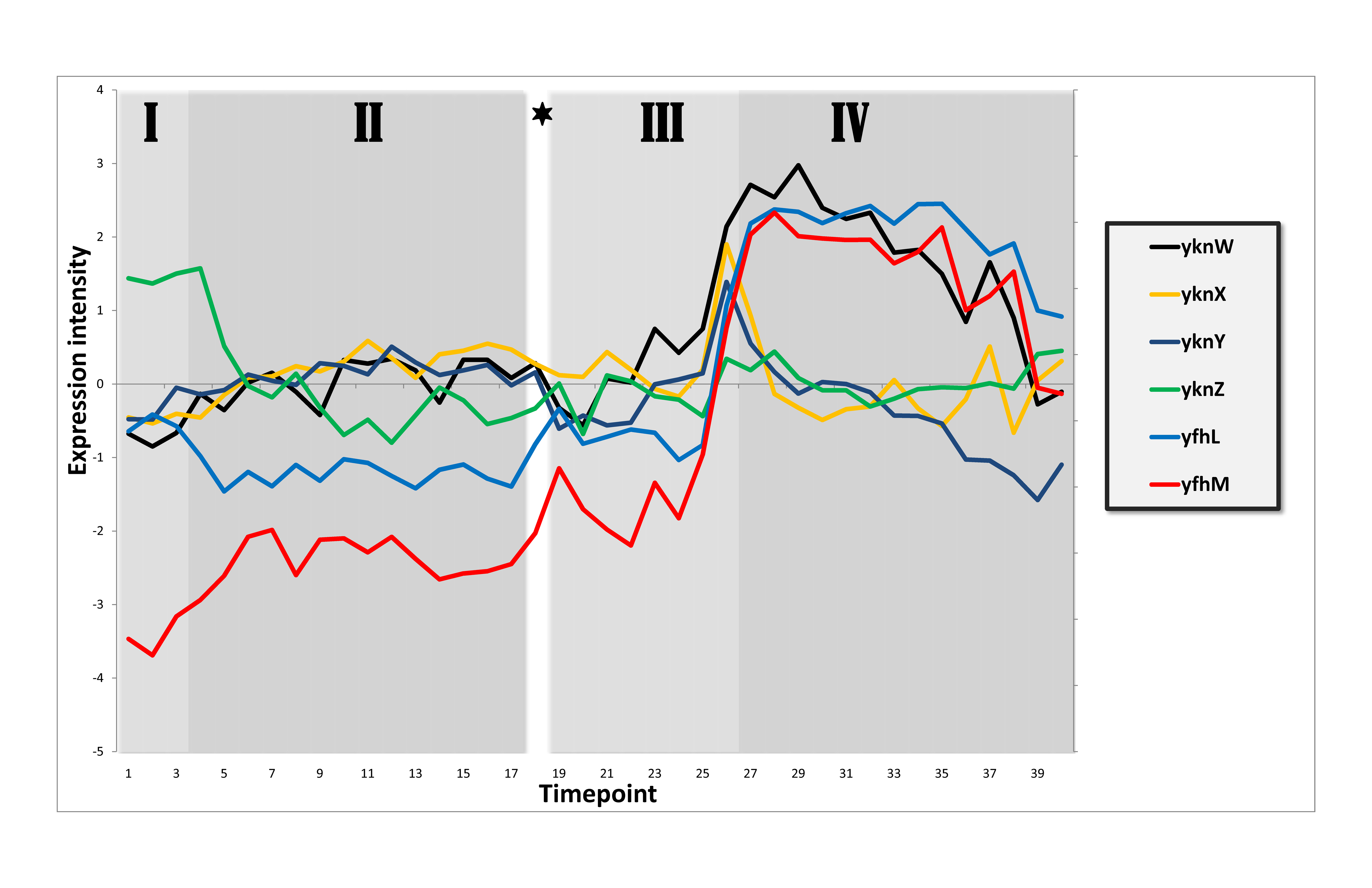

Supplement: Figure S3 — Expression graph of yknWXYZ and yfhLM which mediate SigW dependent resistance to SdpC . (TIF) [file pone.0027160.s003.tif]

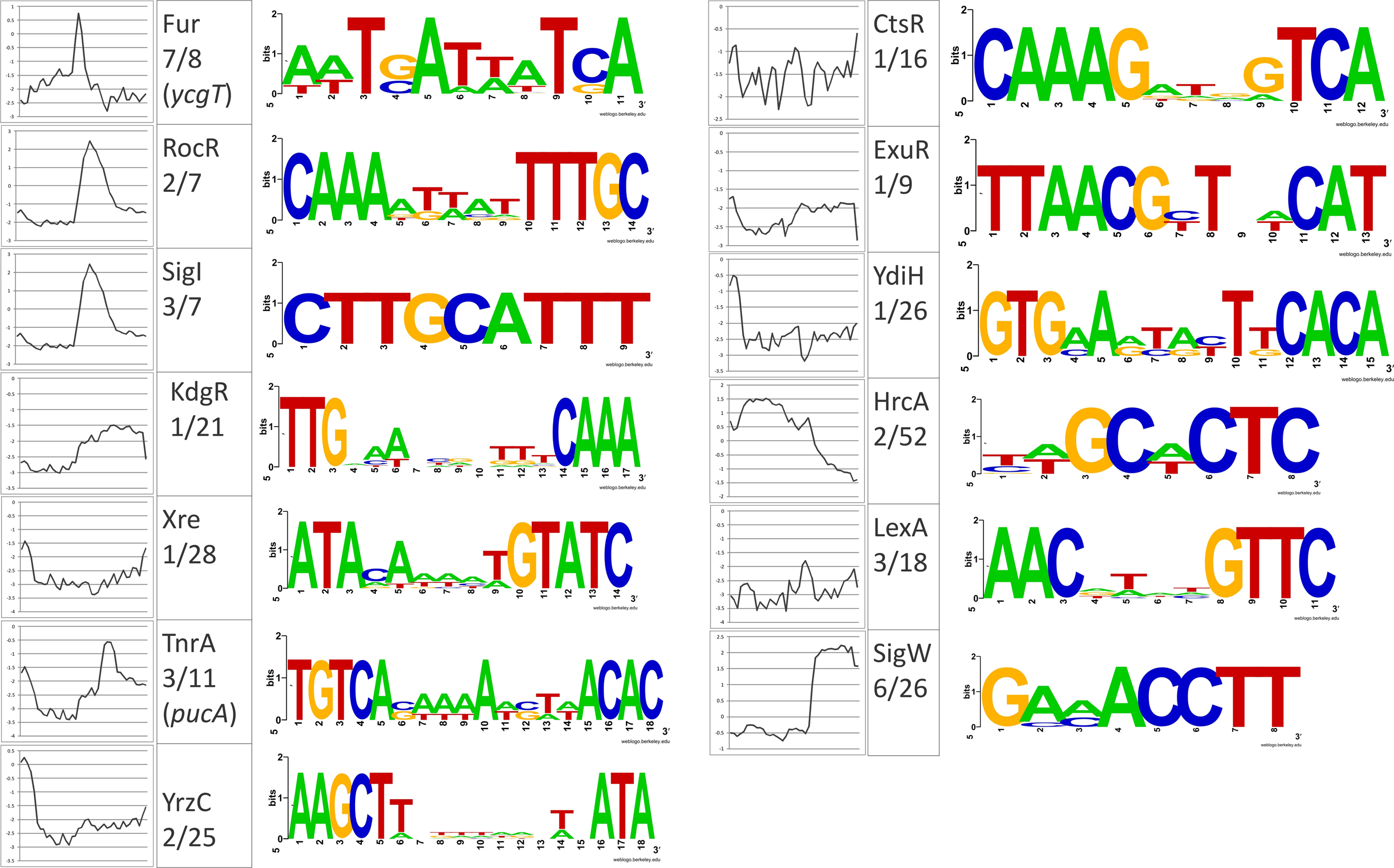

Supplement: Figure S4 — Overview of identified binding sites that overlapped with known binding sites. The first column displays the averaged expression level of the gene members of a cluster. The second column describes the name of the known regulator of which some known binding sites were identified. E.g.; the first cluster consists of 8 operon members of which 7 members contain the Fur binding site in their upstream regions that was identified by DISCLOSE. The identified motifs are visualized as sequence logos in the third column. (TIF) [file pone.0027160.s004.tif]

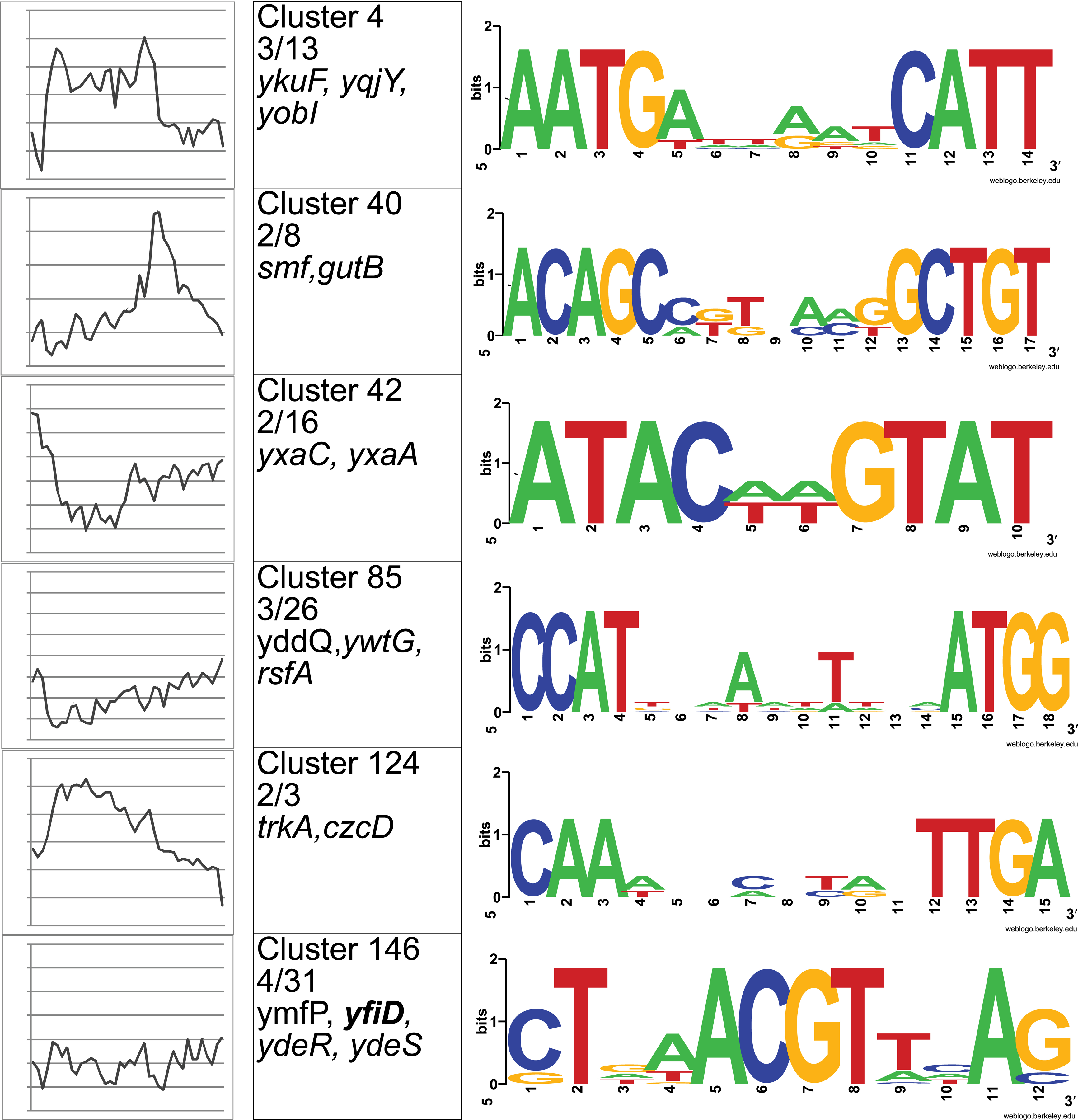

Supplement: Figure S5 — Overview of identified binding sites that do not match with known binding sites. The first column displays the averaged expression level of the gene members of a cluster. The second column describes the name of the cluster, the coverage of the motif and the first genes (genes representing putative or known regulators are indicated in bold) of the operons that contain the motif in their upstream region. E.g.; cluster 4 consists of 13 operons of which 3 members contain a putative binding site in their upstream regions that was identified by DISCLOSE. The identified motifs are visualized as sequence logos in the third column. (TIF) [file pone.0027160.s005.tif]

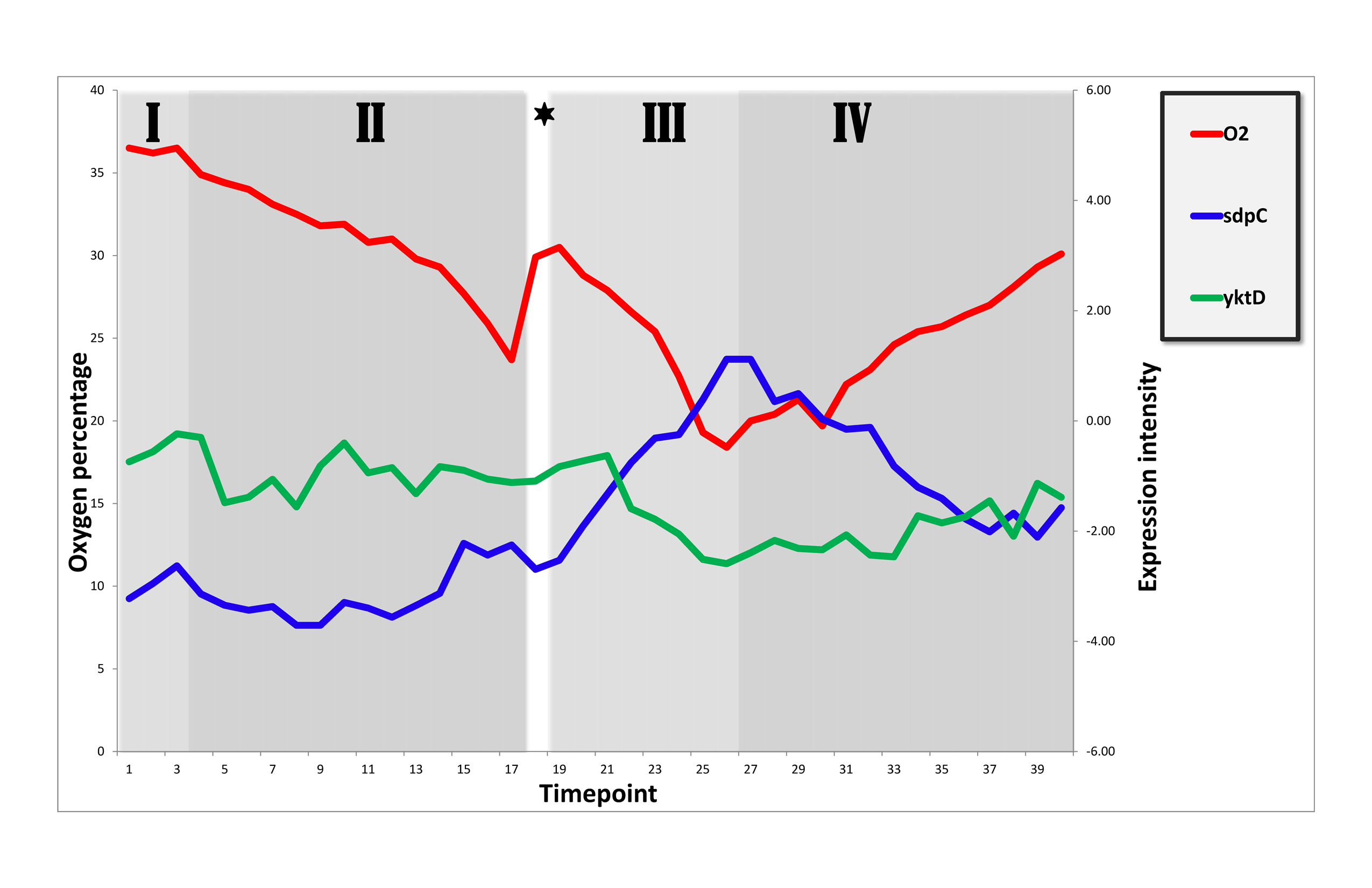

Supplement: Figure S6 — Examples of genes showing correlation (yktD) or anti-correlation (sdpC) with oxygen. The different growth-phases that were identified by the PCA analysis are visualized in the expression graph. I: lag phase, II: exponential growth phase, III: early stationary growth phase. IV: late stationary growth phase. The asterisk represents the transition point. (TIF) [file pone.0027160.s006.tif]
